# Investigation of Lithium Polyacrylate Binders for Aqueous Processing of Ni‐Rich Lithium Layered Oxide Cathodes for Lithium‐Ion Batteries

**DOI:** 10.1002/cssc.202200401

**Published:** 2022-05-03

**Authors:** Friederike Reissig, Sebastian Puls, Tobias Placke, Martin Winter, Richard Schmuch, Aurora Gomez‐Martin

**Affiliations:** ^1^ Helmholtz Institute Münster IEK-12 Forschungszentrum Jülich GmbH Corrensstr. 46 48149 Münster Germany; ^2^ MEET Battery Research Center Institute of Physical Chemistry University of Münster Corrensstr. 46 48149 Münster Germany

**Keywords:** aqueous processing, cathode materials, energy storage, lithium-ion batteries, sustainable chemistry

## Abstract

Ni‐rich layered oxide cathodes are promising candidates to satisfy the increasing energy demand of lithium‐ion batteries for automotive applications. Aqueous processing of such materials, although desirable to reduce costs and improve sustainability, remains challenging due to the Li^+^/H^+^ exchange upon contact with water, resulting in a pH increase and corrosion of the aluminum current collector. Herein, an example was given for tuning the properties of aqueous LiNi_0.83_Co_0.12_Mn_0.05_O_2_ electrode pastes using a lithium polyacrylate‐based binder to find the “sweet spot” for processing parameters and electrochemical performance. Polyacrylic acid was partially neutralized to balance high initial capacity, good cycling stability, and the prevention of aluminum corrosion. Optimized LiOH/polyacrylic acid ratios in water were identified, showing comparable cycling performance to electrodes processed with polyvinylidene difluoride requiring toxic *N*‐methyl‐2‐pyrrolidone as solvent. This work gives an exemplary study for tuning aqueous electrode pastes properties aiming towards a more environmentally friendly processing of Ni‐rich cathodes.

## Graphical abstract

**Sweet spot**: Ni‐rich layered oxide cathodes are promising candidates to satisfy the demand for batteries for automotive applications. Aqueous processing of such materials is challenging due to the Li^+^/H^+^‐exchange in water. Herein, an example is given for tuning the properties of aqueous electrode pastes by varying LiOH/polyacrylic acid ratios as binder and finding the “sweet spot” between processing and electrochemical performance.

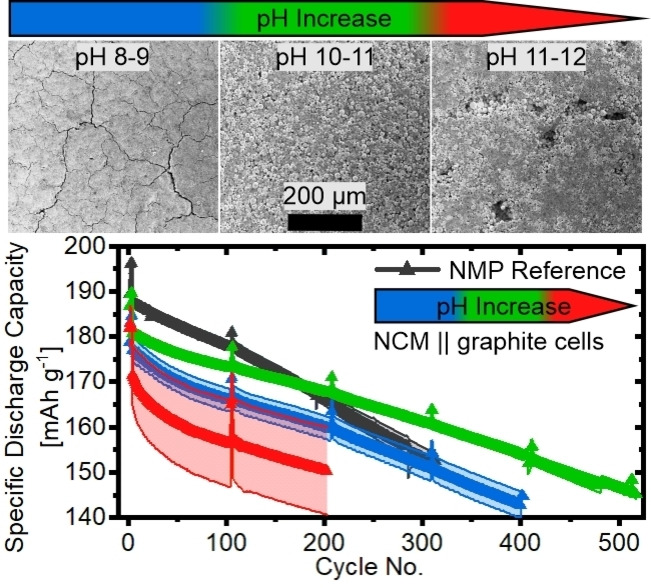

## Introduction

Given the growing concerns about global warming and climate change, there is a huge increase in the demand for renewable energies to reduce greenhouse gas emissions. Along with that comes the need to store the “green” electricity, while every application has significantly different requirements in lifetime, power, cost, and gravimetric and volumetric energy densities. In the private mobility sector, prospective customers will expect comparable cost, safety standards, and driving range of an electric vehicle (EV) compared to a combustion engine‐powered vehicle.^[1]^ Therefore, future generations of battery technologies for EV applications will need to achieve lower cost and presumably higher energy and power density compared to today's standards. Due to their high level of technological maturity combined with a good compromise between energy density, power, energy efficiency, lifetime, and costs, rechargeable lithium‐ion batteries (LIBs) are a prime choice for mobile energy storage, which includes electro‐mobility as the largest future market.[^2^, ^3^, ^4^]

Ni‐rich LiNi_1−*x*−*y*_Co_*x*_Mn_*y*_O_2_ (NCM) layered oxide materials with Ni contents of 60 to 80 % are commercially available cathode active materials to satisfy those needs and therefore enable extensive market penetration of EVs.[^5^, ^6^, ^7^] The main advantage of increasing the Ni content lies in an increased energy density on the material level (higher de‐lithiation capacity at the same charge cut‐off potential) and a reduced content of costly and toxic cobalt.[^8^, ^9^] In addition to material instabilities during cycling, which are currently widely addressed in literature,[^10^, ^11^, ^12^, ^13^, ^14^, ^15^, ^16^, ^17^, ^18^] Ni‐rich NCM is currently processed with toxic organic solvents such as *N*‐methyl‐2‐pyrrolidone (NMP) as state of the art and polyvinylidene difluoride (PVdF) as binder. However, the use of non‐toxic solvents such as water remains challenging due to the Li^+^/H^+^ exchange as soon as NCM is in contact with water and the insolubility of PVdF in water.[^19^, ^20^]

Since aqueous processing of the positive electrode represents a promising approach to make LIBs in mass production more environmentally friendly, two strategies must be pursued: find (i) suitable modification approaches to mitigate the Li^+^/H^+^ exchange and (ii) suitable binder systems in aqueous media compatible with the working potential of cathode materials. Mitigating the Li^+^/H^+^ exchange is important because it leads to two challenges. One is the loss of Li^+^ from the active material, and the second one is the resulting pH increase of the electrode paste leading to a corrosion of the aluminum (Al) foil current collector. On the one hand, the main strategy to mitigate the Li^+^/H^+^ exchange is protecting the cathode particle surface via surface coatings or functionalization^[21]^ since the Li^+^/H^+^ exchange most likely arises from the structural similarity to NiOOH.^[22]^ In addition, suitable binder systems on the other hand are explored for various existing cathode chemistries. While binder combinations from carboxymethylcellulose (CMC), styrene butadiene rubber (SBR), and poly(acrylic acid) (PAA) are widely used for the processing of negative electrodes (anodes), various binders and combinations have been and are currently explored for the use with various cathode chemistries.^[23]^ Examples include, but are not limited to, works with Na‐CMC for LiFePO_4_, high voltage composite cathodes or various NCM stoichiometries,[^24^, ^25^, ^26^, ^27^, ^28^] additives or protective coatings for the Al‐foil,[^29^, ^30^, ^31^] and investigations of the positive effect the natural pH of NCM532 in water^[32]^ and PAA.^[33]^ In addition, for example, an advanced lithium CMC and PAA co‐polymer composite binder indicated promising opportunities for tailoring binder properties for industrial applications.^[34]^ A neutralized LiPAA binder solution, for example, has been reported by Pieczonka et al. as effective binder for LiNi_0.5_Mn_1.5_O_4_ (LNMO) spinel high‐voltage cathodes with the benefit of forming an artificial cathode electrolyte interphase (CEI) layer.^[35]^

In this work, the LiOH/PAA binder system is used for the processing of Ni‐rich NCM cathode materials by aqueous routes. LiOH and PAA react to form lithium polyacrylate, and excess LiOH has to be avoided due to its detrimental effect on the cycling performance via electrolyte decomposition and gas formation.[^36^, ^37^, ^38^] Therefore, a systematic investigation of the influence of the LiOH/PAA ratio on the pH, the electrode pastes’ and electrode properties as well as the cycling stability is reported. As the electrode paste properties will strongly depend on the dispersing device as well as on the pH resulting from the used LiOH/PAA ratio, two different dispersing devices with different working principles are herein evaluated. A purely planetary centrifugal mixer and mild dispersing device (Thinky mixer) is compared with a high‐energy dissolver instrument with a rotating dissolver disc (Dispermat) showing a crucial influence of the dispersing device on the viscosity of the electrode paste. This leads to different optimal LiOH/PAA ratios for both dispersing devices and allows for a long‐term cycling performance comparable to NMP/PVdF reference electrodes. Finally, the effect of isopropanol and ethanol as co‐solvents has been investigated, due to their amphiprotic nature, easy accessibility, good miscibility with water, better evaporation, and the possibility to lower the pH acting on active material, aluminum foil, and mixing devices. The influence of the co‐solvents seems to be of a more complex nature, nonetheless showing a positive impact.

## Results and Discussion

### pH value determination of binder solutions and electrode pastes

**Figure 1:**
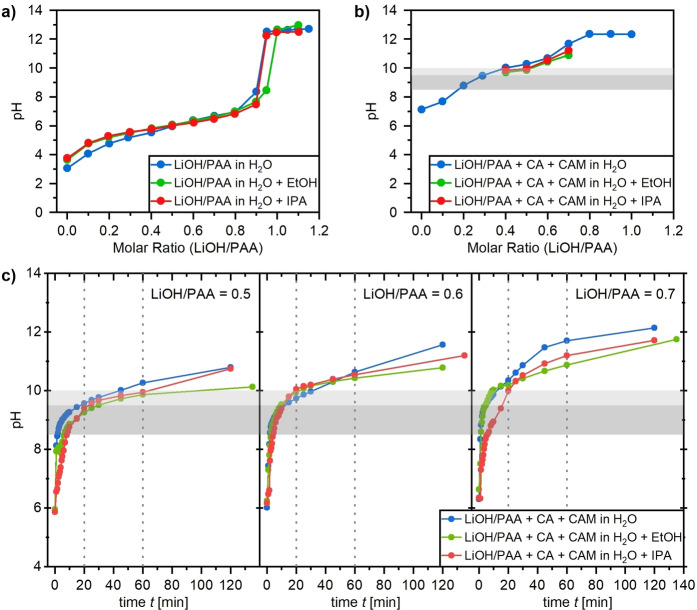
Results of pH measurements. (a) pH of binder solutions with different LiOH/PAA ratios and solvent combinations after proper dissolution of all components. (b) pH of electrode pastes with different LiOH/PAA ratios and solvent combinations after the addition of the CAM to the dispersion with the CA and stirring with the magnetic stirrer for 60 min. (c) Time‐dependent pH measurements after addition of the CAM to the dispersion of LiOH/PAA (0.5, 0.6, and 0.7) and CA and stirring with a magnetic stirrer. Different grey shades mark the range of reported upper pH stability limits of the Al‐foil current collector reported in literature of 8.5 and 10 and the isoelectric point of alumina at pH 9.5.[^40^, ^41^, ^42^]

Systematic pH investigations on LiOH/PAA solutions as well as the electrode pastes were carried out. The LiOH/PAA ratio of the binder system was varied to adapt the electrode paste properties and specially the pH, while pure H_2_O, or combinations with co‐solvents were evaluated. Figure 1 shows pH investigations carried out with a pH meter during or after mixing with a magnetic stirrer. In Figure 1a, the study of solutions with different LiOH/PAA ratios and solvent combinations is shown. The solutions were stirred until all components were dissolved and a stable pH could be measured. For the purely aqueous solution without additional solvents, the half equivalence point (LiOH/PAA=0.5) should correspond to the p*K*_a_ of PAA. The obtained pH value of around 6, however, does not agree with the literature value of p*K*_a_≈4.54.^[39]^ The reason for this difference is that the commercial PAA solution contained 25 wt% PAA that was already neutralized. For the solution LiOH/PAA in H_2_O, there seems to be a reasonably stable pH buffer range between LiOH/PAA ratios of 0.5 to 0.8. This region is slightly extended for both co‐solvents [isopropanol (IPA) and ethanol (EtOH)].

In a next step, the influence of the addition of the conductive agent (CA) and the LiNi_0.83_Co_0.12_Mn_0.05_O_2_ cathode active material (CAM) on the pH of the electrode paste was investigated with regards to the LiOH/PAA ratio or the use of co‐solvents. For all investigations the slurry composition with 50 wt% solid content, split up in 94 wt% CAM, 3 wt% CA and 3 wt% binder, was used. Addition of the conductive agent and stirring for 16 h to ensure good mixing did not result in any change in pH. Afterwards, the CAM was added, and the pH was measured after 1 h of stirring because that is the longest amount of time the CAM is in contact with water during mixing the electrode paste in this work. The obtained pH values are shown in Figure 1b along with the different upper pH stability limits that have been reported in literature for the Al current collector of 8.5 and 10 and the isoelectric point of alumina at pH 9.5 (light and dark grey horizontal bars).[^40^, ^41^, ^42^] Without additional solvents even the highest reported stability limit of pH 10 is reached for a LiOH/PAA ratio of 0.4 with a steep increase of the pH>10 above a ratio of 0.6. Additional solvents (20 wt%) were evaluated for ratios with pH values starting at around 10. The results in Figure 1b indicate that both co‐solvents can lead to a slight pH reduction in this specific experiment, but it has to be noted that the pH is only defined for aqueous media and therefore also only measured based on the aqueous medium. As a general trend it can still be seen that there is no significant effect for the LiOH/PAA ratio of 0.4 but more pronounced effects for the ratios 0.5, 0.6, and 0.7, which will therefore be investigated further.

Time‐dependent pH investigations were carried out on the electrode pastes due to the different contact times of the CAM with water depending on the used dispersion device (20 min for the Thinky Mixer and 60 min for the Dispermat). The time‐dependent pH measurement in Figure 1c was started when the CAM was added to the typical dispersion of LiOH/PAA and the conductive agent in water while everything was continuously stirred with the magnetic stirrer. In all investigated cases (LiOH/PAA=0.5, 0.6, and 0.7; with and without co‐solvent), a logarithmic pH increase with time could be observed. A pH‐lowering effect of the co‐solvents is most noticeable after 1 h. Considering the upper stability limit of pH 10 reported for Al foil, the most promising electrode paste compositions can be chosen for further investigations. For a LiOH/PAA ratio of 0.5 the pH was below pH 10 for most measurements up to 1 h dispersing time, whereas for a ratio of 0.6 this was only true up to 20 min dispersing time. Even though the co‐solvents lowered the pH for the LiOH/PAA ratio of 0.7, the pH was still significantly above 10 for most measurements after 20 min. Therefore, a LiOH/PAA ratio of 0.7 was kept as upper limit of the investigated series, but no co‐solvents samples were investigated further for that ratio.

In addition to proper pH investigations with a pH meter for binder solutions and electrode pastes dispersed by a magnetic stirrer, the pH values were also measured with pH paper prior to casting the electrode sheets after preparation with the Thinky Mixer and the Dispermat dispersing devices. It is important to note that the two different dispersing devices have quite different working principles. While the Thinky Mixer is a purely planetary centrifugal mixer and milder dispersing device without actual tools influencing the mixture, the Dispermat is a high‐energy dissolver instrument with a rotating dissolver disc that enables high shear forces with the possibility to crush agglomerates. The pH values estimated by the use of pH paper for all prepared and investigated electrode compositions can be seen in Table 1. It must be noted that the error range for those measurements is probably quite large (±1) due to the dark color of the electrode paste and the different viscosities, and it is therefore quite subjective, especially in the color range for pH 8–10. While the detailed values need to be treated with care, the overall trend should give an estimation of the pH influence with regard to dispersing device and LiOH/PAA ratio. The samples are labeled with a small letter corresponding to the dispersing device (“*t*‐“ for Thinky Mixer and “*d*‐“ for Dispermat), followed by the molar ratio LiOH/PAA and optionally a capital letter for the co‐solvent used, as shown in Table 2. Co‐solvents could only be used for electrodes prepared with the Thinky Mixer due to the closed containers. For the Dispermat, electrode pastes were prepared in an open container, and therefore it could not be guaranteed that the co‐solvents did not gradually evaporate during the mixing process. As expected, the pH values for the Thinky Mixer and Dispermat electrode pastes slightly differ from the values measured with the pH electrode for the dispersion mixed by the magnetic stirrer. This can be the result not only of the uncertainty of the measurement by a pH paper, but also of the different speeds, times, and mixing mechanism considered for both devices. However, the trends of higher LiOH/PAA ratios resulting in higher pH values are confirmed. In addition, the pH lowering as a result of the addition of the co‐solvents could be further validated and probably results from the amphiprotic character of both solvents. Depending on the literature considered, the threshold for avoiding Al foil corrosion[^41^, ^42^] is reached for LiOH/PAA ratios of 0.4 and 0.6.

**Table 1:** pH values of electrode pastes measured with pH paper for different dispersing devices. Mixing times with the CAM were 20 min for the Thinky Mixer and 60 min for the Dispermat.

Molar ratio LiOH/PAA	pH measured with pH paper prior to coating			
	Thinky Mixer			Dispermat
	H_2_O [*t*‐__]	H_2_O+EtOH [*t*‐__‐E]	H_2_O+IPA [*t*‐__‐I]	H_2_O [*d*‐__]
0.0	6–7	–	–	6–7
0.1	7	–	–	–
0.2	7–8	–	–	–
0.3	8	–	–	–
0.4	8–9	7	7	8
0.5	10	9	9–10	8–9
0.6	10–11	8–9	9	11
0.7	11	–	–	11–12

**Table 2:** Overview over the prepared positive electrode pastes.

Molar ratio LiOH/PAA	Sample names			
	Thinky Mixer			Dispermat
	H_2_O	H_2_O+EtOH	H_2_O+IPA	H_2_O
0.0	*t‐*0.0	–	–	*d‐*0.0
0.1	*t‐*0.1	–	–	–
0.2	*t‐*0.2	–	–	–
0.3	*t‐*0.3	–	–	–
0.4	*t‐*0.4	*t‐*0.4‐E	*t‐*0.4‐I	*d‐*0.4
0.5	*t‐*0.5	*t‐*0.5‐E	*t‐*0.5‐I	*d‐*0.5
0.6	*t‐*0.6	*t‐*0.6‐E	*t‐*0.6‐I	*d‐*0.6
0.7	*t‐*0.7	–	–	*d‐*0.7
NMP/PVdF‐reference	*t‐*Ref	–	–	*d‐*Ref

### Analysis of Al current collector corrosion

**Figure 2:**
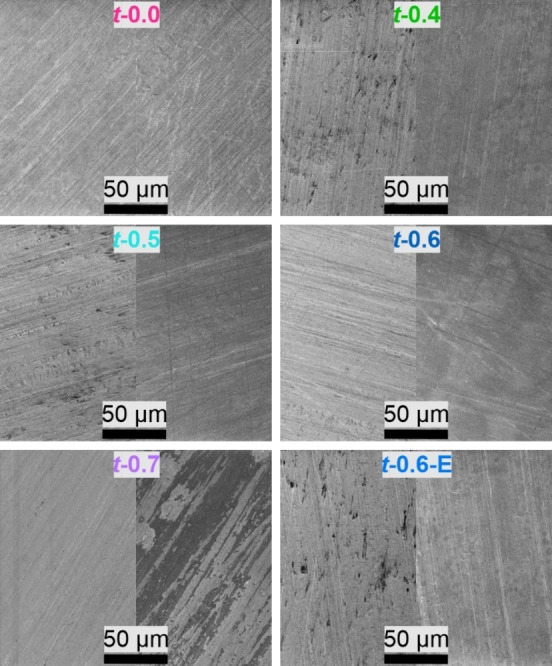
SEM images of Al foil current collector after cleaning the surface with EtOH and after contact with selected electrode pastes prepared by the Thinky Mixer. The left part for each ratio shows the pristine Al foil only cleaned with EtOH. The right part for each ratio shows the Al foil after cleaning with ethanol, contact with the respective electrode paste for 4–5 min followed by removing the electrode paste. The samples are labeled with a small letter corresponding to the dispersing device (“*t*‐’’ for Thinky Mixer), followed by the molar ratio LiOH/PAA and optionally a capital letter for the co‐solvent used.

In a next step, the actual influence of the electrode paste on the Al foil corrosion was investigated via scanning electron microscopy (SEM) of Al foil (Figure 2) that was either only cleaned with ethanol (left images) or was in contact with the electrode paste for 4–5 min before it was removed (right images). This time corresponds to the time before the electrode paste started drying at the edges of the electrode sheets. After that time, the Al foil surface looked slightly etched for all electrode pastes apart from *t*‐0.0, which corresponds to a pH of 6–7. However, only the electrode paste of *t‐*0.7 (pH≈11) leads to a significantly more inhomogeneous Al foil surface appearance. As can be seen, this effect is less pronounced for *t‐*0.6, where the Al foil looks slightly unevenly etched. For the remaining ratios *t*‐0.4 and *t‐*0.5 as well as for the electrode paste with ethanol addition *t‐*0.6‐E, the Al foil surface looks smoother and even more homogeneous after the slurry contact. This might result from etching the Al_2_O_3_ layer off the Al foil surface. However, slight scratches and inhomogeneity are present throughout all measurements resulting from the manufacturing process of the Al‐foil and the cleaning process with ethanol‐soaked tissues.

### Rheology studies of electrode pastes

Rheological properties of electrode pastes are important to ensure homogeneous mixing and at the same time a stable electrode paste during transfer, coating, and drying process. Therefore, a shear thinning behavior (i. e., low viscosities at high shear rates and high viscosities at low shear rates) is required.[^43^, ^44^] For the Thinky Mixer it was possible to measure all electrode pastes with the used measuring geometry that is suitable for the particle sizes in the cathode paste (parallel plate geometry with a gap of at least 10× the particle size). In contrast, the electrode pastes prepared with the Dispermat with LiOH/PAA ratios below 0.6 were not sufficiently viscous to be properly measured.

**Figure 3:**
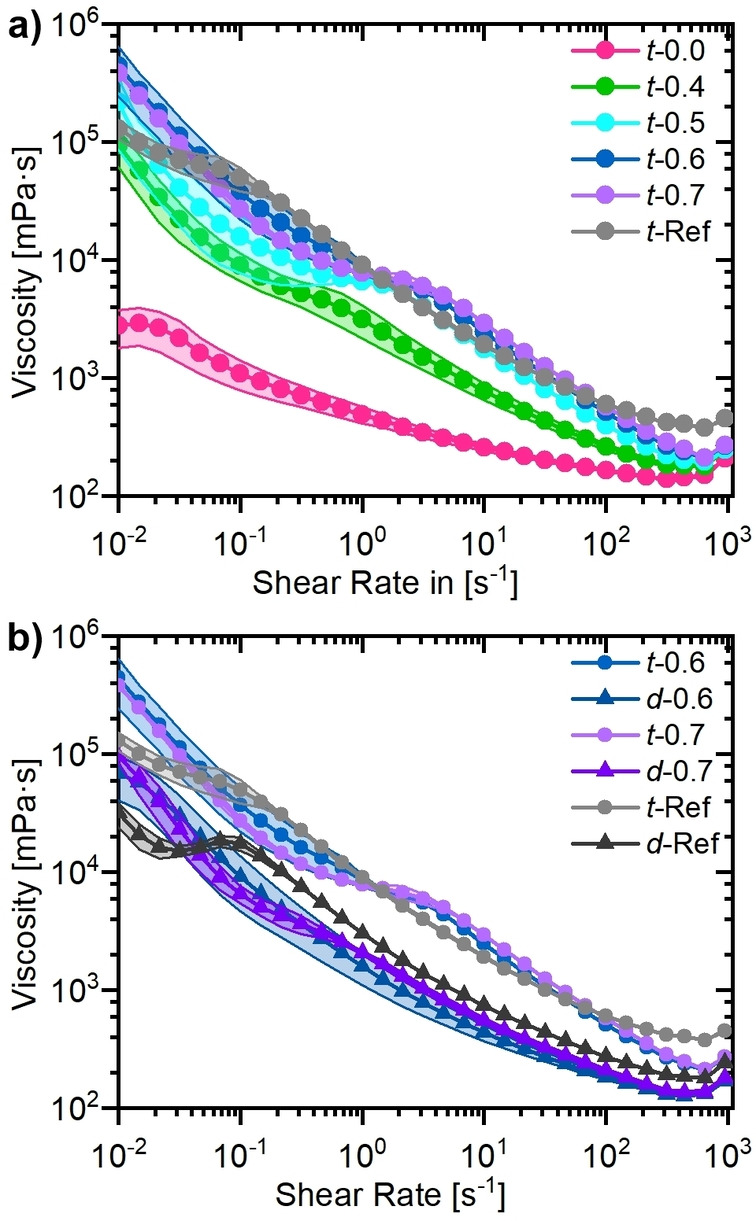
Rheological properties of selected cathode pastes. (a) Impact of electrode pastes with various LiOH/PAA ratios prepared via the Thinky Mixer in comparison to PVdF/NMP pastes (*t*‐Ref). Additional LiOH/PAA ratios can be found in Figure S1. (b) Comparison of the impact of the different processing methods Thinky Mixer (*t*‐) and Dispermat (*d*‐) including PVdF/NMP electrode pastes as reference (‐Ref). The samples are labeled with a small letter corresponding to the dispersing device (“*t*‐’’ for Thinky Mixer and “*d*‐’’ for Dispermat), followed by the molar ratio LiOH/PAA and optionally a capital letter for the co‐solvent used.

As shown in Figure 3 and Figure S1, all electrode pastes fulfill the requirement of low viscosities at high shear rates and are comparable in some cases to the respective NMP/PVdF reference electrode pastes. Figure 3a and Figure S1a show how the rheological behavior of the electrode pastes changes with the LiOH/PAA ratio. Electrode pastes with more LiOH such as *t‐*0.7, *t*‐0.6, and *t*‐0.5 have higher viscosities at low shear rates, which are comparable to the one of *t*‐Ref even though the course of the flow curves differs. Moving from there to lower LiOH/PAA ratios, a constant lowering of the viscosity at low shear rates from *t*‐0.4 to *t*‐0.0 can be observed. This suggests that the viscosity differences are related to different pH values of the electrode pastes. The pH value will increase with enhanced LiOH/PAA ratio. Higher pH values imply that the carboxyl groups of the PAA chains are deprotonated, which should result in a repulsion of the carboxyl groups. In literature, the PAA chains were found to be tightly coiled at low pH values and have a more extended conformation at higher pH values.^[45]^ However, the different other components of the electrode pastes, the CAM, and the CA, will have an impact on the arrangement of the PAA chains, thus, making the system much more complex to understand. Sung et al. investigated the influence of the pH on the viscosity of suspensions consisting of PAA and CA (Conductex 7067 Ultra, Birla carbon) and/or graphite (SG‐BH8, Ito Graphite Co., Ltd.). They found polymer–particle interactions to cause the rheological differences, as well as a pH‐dependent adsorption of PAA on graphite particles.^[46]^ The herein discovered trend of more viscous cathode electrode pastes with increasing pH values was also observed for those graphite pastes.^[46]^

For a comparison of the impact of the dispersing device on the rheological properties, data from Dispermat and Thinky Mixer electrode pastes are compared in Figure 3b. It is remarkable that all electrode pastes prepared by the Dispermat show a significantly lower viscosity for both aqueous PAA and NMP/PVdF electrode pastes. Possible origins for that might be either different binder chain conformations due to the different mixing methods or that the high energy or sharp edges of the Dispermat dissolver disc might shorten the polymer chains. Investigations via differential scanning calorimetry (DSC) were performed to determine the glass transition temperatures of the pristine polymer and after 1 h treatment with one of the dispersing devices. The results can be found in Figure S2 and Table S1 and indicate that there is indeed a change in the glass transition temperature depending on the mixing device. The glass transition temperature is generally decreased after the Dispermat treatment compared to the pristine material, while it is increased after the Thinky Mixer treatment. Since it seems unlikely that the Thinky Mixer leads to the formation of longer PAA chains it is thus more likely that this change in glass transition temperature originates from a different coil conformation. The decreased glass transition temperature after the Dispermat treatment could result either from shortened chains or again from another coil conformation.

Finally, also the influence of the co‐solvents (EtOH and IPA) on the electrode paste properties is investigated (Figure S1b). For *t*‐0.5 and *t‐*0.6 the samples with co‐solvent substitution *t‐*0.5‐E, *t‐*0.5‐I, *t‐*0.6‐E, and *t‐*0.6‐I show similar rheological properties and there are only changes within the error range of the samples. However, for *t‐*0.4 the co‐solvents lead to the desired higher viscosities at low shear rates for *t‐*0.4‐E and *t‐*0.4‐I. This demonstrates an additional interesting influence of the co‐solvents, which seem to not only lower the pH of the paste but at the same time also improve the viscosity.

### Impact of aqueous processing on electrode structure and properties

**Figure 4:**
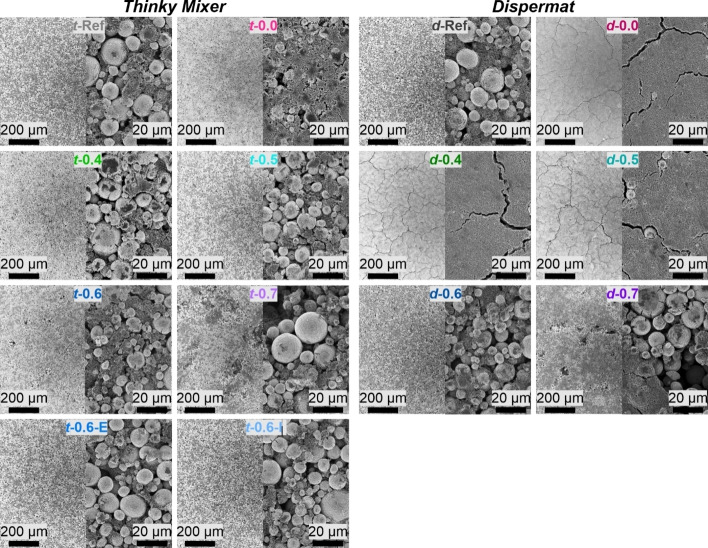
SEM images of composite cathodes prepared by different processing methods (Thinky Mixer and Dispermat) at two different magnifications. The samples are labeled with a small letter corresponding to the dispersing device (“*t*‐’’ for Thinky Mixer and “*d*‐’’ for Dispermat), followed by the molar ratio LiOH/PAA and optionally a capital letter for the co‐solvent used (I stands for IPA and E for EtOH).

SEM measurements were also performed to determine the impact of the aqueous processing on the resulting electrode surface morphology. All aqueous processed LiOH/PAA electrodes showed an improved adhesion (126–192 N cm^−2^) to the current collector compared to the PVdF/NMP‐based reference electrodes (83 N cm^‐2^) after calendering. This is in agreement with previous observations for long PAA chain lengths (*M*≥450000 g mol^−1^) by Bauer et al.^[42]^ However, there are significant differences in the top view in the SEM as shown in Figure 4. While *t*‐Ref and *d*‐Ref electrodes both show a homogeneous distribution of all electrode components, this is not the case for all aqueously processed electrodes.

For lower LiOH/PAA ratios (i. e., electrode pastes that either could not be measured in the rheological measurements or showed viscosities below ≈10^4^ mPa⋅s) there is significantly more CA visible at the electrode surface, as shown in Figure 4 and Figure S3. This suggests a sedimentation of the denser CAM particles, while the less dense CA particles float on top. For very low viscosities such as *d*‐0.5, *d*‐0.4, and *d*‐0.0, which could not be measured in the rheological measurements, this sedimentation was probably so pronounced that there are almost no CAM particles visible at the electrode surface. This results in a severe cracking of the electrode surface after drying, as visible in the respective SEM images.

For higher LiOH/PAA ratios, a too alkaline pH (>10) and therefore Al corrosion leads to the formation of pits in the composite electrode surface, which most likely arise from H_2_ gas evolution. While the pits are similar in size and pronounced for *t*‐0.7 and *d*‐0.7, this is not the case for *t*‐0.6 and *d*‐0.6. Electrode *t*‐0.6 shows pits in the electrode surface while *d*‐0.6 does not, even though *d*‐0.6 has a higher pH value. The reason for that might lay in the lower viscosity of the Dispermat electrode pastes (as reported in Figure 3), which allows the formed gas to escape more easily and is then able to flow back in position afterwards. For *t*‐0.6, the co‐solvents can prevent the pit formation probably due to the lower pH affecting the Al foil corrosion since all electrode pastes had the same rheological properties. As visible in Figure 4 and Figure S3, 25 wt% of EtOH or IPA as co‐solvents also do not lead to any arising challenges upon drying of the electrode sheets.

### Electrochemical characterization of aqueously processed electrodes

Various electrochemical investigations were performed on the aqueously processed electrodes as well as NMP/PVdF reference electrodes. Linear sweep voltammetry (LSV) was performed in a three‐electrode NCM||graphite cell with a Li metal reference^[47]^ on selected samples, as shown in Table S2. It could be shown that comparable or even better oxidative stability can be obtained for the aqueously processed electrodes compared to the NMP/PVdF references.

In addition, rate capability studies of aqueously and NMP‐processed electrodes were performed in two‐electrode NCM||Li metal cells to avoid Li metal plating on graphite electrodes^[48]^ and separate the influence of the anode material on the overall performance. The C‐rate was therefore only varied upon discharge and kept at 0.2 C during charge to minimize inhomogeneous Li metal plating [i. e., formation of high‐surface‐area lithium (HSAL)]^[49]^ on the Li metal anode happening in state‐of‐the‐art organic carbonate‐based electrolytes.^[50]^ The data are shown in Figure S4. Discharge capacities with 4.3 V as upper cut‐off voltage at 0.1 C range between 185–205 mAh g^−1^ with the highest attainable discharge capacities obtained for the NMP‐processed reference electrodes. All aqueous Dispermat electrodes outperform the corresponding NMP‐based reference electrodes with regard to rate capability, and they show higher capacities at higher rates (158–168 mAh g^−1^ compared to 155 mAh g^−1^ at a discharge rate of 3 C), while the contrary can be observed for the electrodes prepared with the Thinky Mixer (158–167 mAh g^−1^ compared to 173 mAh g^−1^ at a discharge rate of 3 C). However, within the respective sets of samples there are no systematic differences visible, and the aqueously processed electrodes seem to perform more similarly for the two dispersing methods than to the NMP/PVdF references.

Long‐term charge/discharge cycling experiments were performed in NCM||graphite cells until 80 % state‐of‐health (SOH) was reached (SOH with reference to the 1st discharge capacity at a rate of 0.33 C, corresponding to cycle No. 5). Four formation cycles were conducted at 0.1 C (20 mA g^−1^) to allow reliable comparison between datasets,^[51]^ while the following long‐term cycling took place at 0.33 C with 2 recovery cycles at 0.1 C every 100 cycles. Table S3 shows the initial coulombic efficiencies (*C*_Eff_), initial discharge capacities at 0.1 and 0.33 C after formation and the cycle where the pre‐defined end‐of‐life has been reached for all cells.

**Figure 5:**
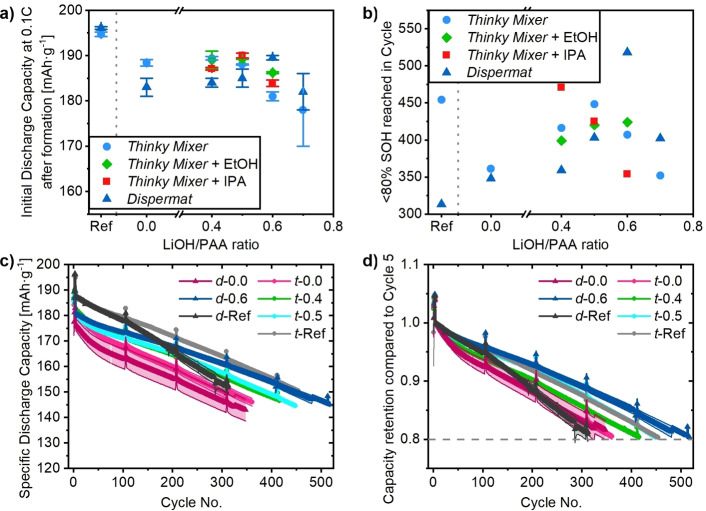
Results of electrochemical characterization in NCM||graphite full‐cells with 4.2 V upper cut‐off voltage. The first four cycles were conducted at 0.1 C (1 C=200 mA g^−1^), while the following long‐term cycling took place at 0.33 C with two cycles at 0.1 C every 100 cycles. (a) Initial discharge capacities at 0.1 C after formation vs. the LiOH/PAA ratio compared to the PVdF/NMP references (Ref). (b) Cycle in which the end‐of‐life criterion of 80 % SOH is met vs. the LiOH/PAA ratio with the PVdF/NMP references in comparison. (c) Specific discharge capacities vs. cycle number of the PVdF/NMP references, the best‐performing LiOH/PAA ratio per dispersing device and the PAA paste without LiOH. (d) Capacity retention calculated based on the capacity achieved in cycle 5 as SOH determination, for the PVdF/NMP references, the best‐performing LiOH/PAA ratio per dispersing device and the PAA pastes without LiOH. The samples are labeled with a small letter corresponding to the dispersing device (“*t*‐’’ for Thinky Mixer and “*d*‐’’ for Dispermat), followed by the molar ratio LiOH/PAA and optionally a capital letter for the co‐solvent used.

Results of the electrochemical characterization in NCM||graphite full‐cells with 4.2 V as upper cut‐off voltage are shown in Figure 5, while additional samples can be found in Figure S5 and S6 with summarized electrochemical performance data in Table S3. The initial discharge capacities at 0.1 C after formation are presented in Figure 5a and show that all LIB cells based on aqueous electrode pastes have a lower initial discharge compared to the NMP references. As the anode material and cell assembly conditions were the same, differences between cells most likely result from differences in the positive electrode. Most cells containing positive electrodes processed aqueously show similar initial discharge capacities apart from *d*‐0.7, *t*‐0.7, and *t*‐0.6, which all showed pits in the electrode surface due to the high pH of the electrode paste during its processing, which is probably the cause of the lower initial capacity. Each set of samples has an increasing initial discharge capacity with increasing LiOH/PAA ratio up to a threshold where it drops again. For the Dispermat the optimum is *d*‐0.6 while the optimum for the Thinky Mixer is around *t*‐0.4 and *t*‐0.5. The same is true for the Thinky Mixer electrode pastes with co‐solvents where *t*‐0.4‐E, *t*‐0.5‐E, and *t*‐0.5‐I show the best initial discharge capacities.

First cycle *C*_Eff_ are around 86–87 % for NMP‐processed electrodes and 82–85 % for aqueously processed electrodes with most samples ranging between 84–85 %. The lower first cycle *C*_Eff_ probably results from the Li^+^ loss during the aqueous processing of the Ni‐rich cathode material. During long‐term cycling, the *C*_Eff_ of all cells is increasing from 99.8 to >99.9 % in the first 50 cycles while continuing >99.9 % until the end‐of‐life is reached.

Figure 5b shows the cycle in which the end‐of‐life criterion of 80 % SOH is reached depending on the LiOH/PAA ratio. Each dispersing device series without co‐solvent shows an improved cycling stability with higher LiOH/PAA ratio with a maximum at the last electrode paste composition that did not lead to pits in the electrode surface, followed by a sharp decrease at increased ratios. These cycle life maxima are obtained for *t*‐0.5 after 448 cycles (compared to 454 cycles for *t*‐Ref) and *d*‐0.6 after 518 cycles (compared to 313 cycles for *d*‐Ref). For the compositions with co‐solvent addition, the cycling stability with more LiOH increases for the paste with ethanol, while it decreases for the paste containing isopropanol. The maxima are therefore reached for *t*‐0.4‐I with 471 and *t*‐0.6‐E with 424 cycles. In Figure 5c, the specific discharge capacities of the NMP/PVdF references are compared to the PAA samples without LiOH addition and with the best performing LiOH/PAA samples for the respective dispersing device. Figure 5d shows the capacity retention for the same datasets. The first interesting observation is that *d*‐Ref has a similar performance to *t*‐Ref for the first 100 cycles but experiences a significantly stronger capacity fading thereafter. However, the reason for that is unknown at this point, but the issue could be observed throughout different experiments. The PAA samples without LiOH addition (*t*‐0.0 and *d*‐0.0) show lower initial capacities and a stronger capacity fading within the error range of the other dispersing device. *t*‐0.4 and *t*‐0.5 show similar discharge capacities from cycle 50 onwards, but due to their slightly different initial capacities that result in different SOH. *d*‐0.6 starts at a lower capacity than the NMP references but outperforms *d*‐Ref after 170 cycles and even catches up in capacity to *t*‐Ref due to better cycling stability.

An additional aspect to be mentioned is the polarization growth during cycling that can be estimated via the difference between charge and discharge mean voltages (Δ*V*) shown in Figure S7. The lowest polarization growth is observed for the NMP *d*‐Ref and *t*‐Ref. All samples where the electrode surfaces showed no visible pits or signs of corrosion exhibit a slightly higher polarization growth. A significantly (i. e., 3–4 times) higher polarization is only observed for the electrodes that showed visible Al current collector corrosion and pits at the composite electrode surface (*d*‐0.7, *t*‐0.7, and *t*‐0.6).

## Conclusion

In this work, aqueous processing of a Ni‐rich layered oxide cathode material with a binder system consisting of LiOH and PAA was systematically investigated. Different molar ratios of LiOH/PAA showed the ability to adjust the pH value of electrode pastes. Conventional Al foil was used as current collector without any protective coatings, and it was found that significant inhomogeneous Al corrosion occurs above a pH of 10. In addition, slight Al foil corrosion does not cause significant negative influences on the electrochemical performance if there is no extended pit formation at the surface. Instead, slight etching simply cleans the Al foil by probably (partially) removing the native Al_2_O_3_ layer. The electrode pastes viscosities and rheological properties strongly depend on the pH, the used dispersing device as well as the addition of possible co‐solvents. High‐energy mixing with the Dispermat device and/or low LiOH/PAA ratios generally resulted in less viscous electrode pastes, which led to an inhomogeneous distribution of the electrode pastes components upon drying and, hence, a worsened electrochemical performance (i. e., capacity retention). It is therefore possible and important to adjust the pH and the electrode paste composition to the respective dispersing device to obtain the desired electrode paste properties. All aqueous electrodes showed a sufficiently high adhesion and electrochemical stability window with and without addition of co‐solvents during electrode manufacturing.

It can be concluded that aqueously processed electrodes can show a long‐term cycling stability that is comparable to electrodes prepared with NMP and PVdF as state‐of‐the‐art materials. However, a remaining challenge is the lower initial discharge capacity of aqueously processed electrodes in comparison to the NMP‐processed ones. The cycling stability can be improved by careful tuning of the electrode paste properties via pH adjustment. For the PAA used for this work and the specific dispersing devices the best long‐term cycling performances were obtained with a LiOH/PAA ratio of 0.5 for the electrode prepared by the Thinky Mixer and 0.6 for the ones prepared by the Dispermat. In addition to that, it could be shown that co‐solvents are suitable to enable a lower pH of the electrode pastes and improve the electrochemical performance without negative side effects. Herein it was demonstrated that careful systematic investigations of the binder system as well as the dispersing devices are needed on the route towards more environmentally friendly processing of Ni‐rich LiNi_1−*x*−*y*_Co_*x*_Mn_*y*_O_2_ cathode electrodes for high‐energy lithium‐ion battery cells.

## Experimental Section

### Material characterization

pH measurements were performed with a pH‐electrode (SJ 113, VWR International, LLC.) and a handheld pH meter (pH 20, VWR International, LLC.). Three different types of pH measurements were performed. Firstly, a pH titration curve of PAA (25 wt% PAA solution in water; *M.W*.≈240000; Alfa Aesar) with LiOH⋅H_2_O (Fischer Scientific) was recorded. For that, a stock solution was prepared by dissolving the 25 wt% PAA solution (5.02 g) in distilled water (50 mL). Different amounts of LiOH⋅H_2_O were dissolved each in 3 mL of the stock solution, obtaining solutions with molar ratios of LiOH/PAA between 0 and 1.2 in steps of 0.1. For each solution, the pH value was measured. Secondly, the pH values of the electrode pastes at different LiOH/PAA ratios were investigated. The CA (Super C65, Imerys Graphite & Carbon; 0.09 g) was added to these solutions and stirred overnight. Afterwards, the pH was measured. The CAM (2.83 g) was added to the solutions and after 1 h, the pH of the obtained electrode paste was measured. Thirdly, the evolution of the pH value over time during dispersion was recorded. This was done by placing each PAA stock solution (10 mL) into a vial. After adding and dissolving of different amounts of LiOH⋅H_2_O in these solutions, the CA (0.26 g) was added, and everything was stirred overnight. Afterwards, the pH electrode was attached, and the pH was measured. The CAM (7.99 g) was added and after specific times, the pH was noted. The measurements were performed for the LiOH/PAA ratios 0.5, 0.6, and 0.7, each with 25 wt% EtOH, 25 wt% IPA, or without any co‐solvent. For the pH measurements with solvents, 25 wt% of the water in the stock solution (12.5 g) was replaced by EtOH or IPA. For pH measurements after mixing with the Thinky Mixer (THINKY U.S.A., INC.) or the Dispermat (LC30, VMAGetzmann GmbH, dissolver disk Ø=20 mm) as described below universal pH indicator paper pH 0–14 from Supelco® Merck KGaA was used.

For the rheology measurements, the electrode pastes were prepared with the Dispermat or Thinky Mixer, respectively, as described below. The prepared electrode pastes (1 mL) were analyzed using a MCR 301 Rheometer (Anton Paar Group AG). Three flow curves were recorded for each sample. The standard deviation is represented by error bars in the figures. The shear rate was increased from 10^−2^ to 10^3^ s^−1^. A parallel‐plate (PP50) geometry was used as the measuring system. During the measurement, a solvent trap was used.

Aluminum corrosion was investigated for the various LiOH/PAA ratios. For that, electrode pastes with LiOH/PAA‐ratios between 0.0 and 0.7 were prepared with the Thinky Mixer as described below. They were coated with a doctor blade gap of 100 μm to the aluminum foil. After five minutes of drying at 70 °C, the coating was wiped off the aluminum foil by means of Kimtech Science wipes and distilled water. The sheets were dried overnight at 70 °C. Afterwards, 1 cm^2^ pieces were cut from the coating edge, containing an area that was not covered by the coating and an area that was covered by the coating. They were attached to a carbon pad and transferred into the SEM.

The adhesion measurements were performed on a Zwicki universal testing machine (ZwickRoell GmbH & Co. KG) and with the software testXpert II. Electrode sheets were prepared as described below. Afterwards, the sheets were fixed with a double‐sided tape (Flooring Tape Extra Strong Hold 5696, Tesa SE) onto the metallic plate on the bottom of the device. The plate on top was covered only with double‐sided tape. Top and bottom part were pressed together for 60 s with 2000 N. The maximum traction, which was needed to remove the coating from the aluminum foil, was measured by the device. Five measurements per sample were performed to ensure reproducibility of the results.

The Al foil corrosion and electrode surfaces were investigated by SEM analysis using a Carl Zeiss AURIGA field emission microscope with a Schottky field emitter as electron source. The typical accelerating voltage was 3 kV. Al foil corrosion was investigated for electrodes cleaned after 4–5 min of contact with the electrode paste while the references were only cleaned with ethanol. Electrode surfaces were investigated after calendering of the electrodes.

### Electrode preparation

**Figure 6:**
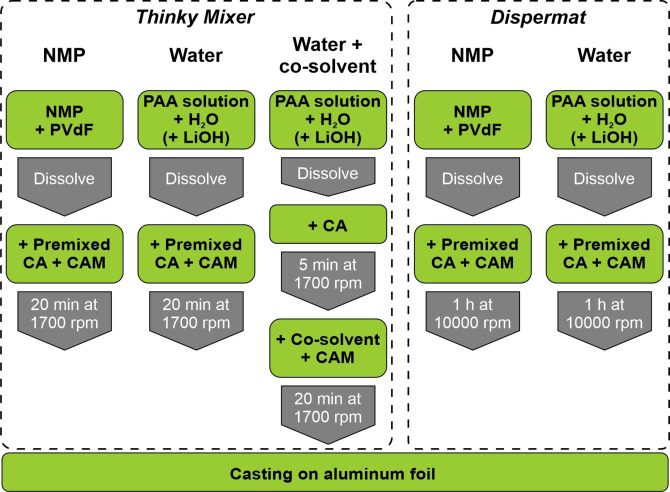
Processing chart of the electrode paste preparation. The Ni‐rich positive electrodes consisted of 94 wt% CAM LiNi_0.83_Co_0.12_Mn_0.05_O_2_ (NCM‐831205), 3 wt% of carbon black as CA and 3 wt% PVdF binder for the non‐aqueous processing and PAA (25 wt% PAA solution in water; *M.W*.≈240000) with optional LiOH⋅H_2_O for the aqueously processed electrodes.

As shown in Figure 6, the Ni‐rich positive electrodes consisted of 94 wt% CAM LiNi_0.83_Co_0.12_Mn_0.05_O_2_ (NCM‐831205; “S85EL‐product”, Ronbay Technology, China), 3 wt% of carbon black as CA (Super C65, Imerys Graphite & Carbon), and 3 wt% PVdF binder (Solef 5130, Solvay) for the non‐aqueous processing and PAA (25 wt% PAA solution in water; *M.W*.≈240000; Alfa Aesar) for the aqueously processed electrodes. Two different mixing methods were used to form a homogenous electrode paste: firstly, the Thinky Mixer ARE‐310 (THINKY U.S.A., INC., samples made with this method referred to as *t*‐), and secondly the Dispermat LC30 (VMAGetzmann GmbH, samples referred to as *d*‐).

For the non‐aqueously processed electrodes, PVdF (0.15 g) was dissolved in NMP (anhydrous, purity: 99.5 %, Sigma‐Aldrich, 5 g). Conductive agent (0.15 g) and CAM (4.7 g) were added to the solution. The electrode paste mixed by the Thinky Mixer (*t‐*Ref) was mixed for 20 min at 1700 rpm. The Dispermat‐mixed electrode paste (*d‐*Ref) was stirred for 1 h at 10,000 rpm with a dissolver disk with Ø=20 mm.

For the aqueously processed electrodes, the PAA solution (0.5915 g) was diluted with distilled water (4.57 mL). Different amounts of LiOH⋅H_2_O (Fisher Scientific) were added to the solution to obtain certain molar ratios of LiOH/PAA, varying between 0.0 and 0.7, to form lithium polyacrylate binders.^[52]^ This ratio is included in the second half of the sample name. For the Thinky Mixer aqueous electrode pastes, the conductive agent (0.15 g) was added to the solution together with the CAM (4.7 g). The suspension was mixed for 20 min at 1700 rpm. For the second method using the Dispermat, the conductive agent and the CAM were added to the aqueous LiOH/PAA solution, and the resulting suspension was stirred for 1 h at 10,000 rpm.

For the aqueous electrodes with co‐solvents, 25 wt% (1.18 g) of the water was replaced by ethanol (EtOH, BASF) or IPA (BASF). For these electrodes, the PAA solution was diluted with distilled water (3.4 g) and different amounts of LiOH⋅H_2_O were added. Afterwards, the CA (0.15 g) was given to the solution and the formed suspension was mixed for 5 min at 1700 rpm in the Thinky Mixer. Then, IPA or EtOH (1.18 g; 25 wt% of the total amount of solvents) was dripped to the suspension, the CAM (4.7 g) was added, and everything was mixed for 20 min at 1700 rpm.

After complete dispersion, the pastes were coated on aluminum foil (20 μm, Nippon foil, previously washed with ethanol) using a doctor‐blade (Zehntner GmbH) and an automatic film applicator (Sheen Instruments) with doctor blade gap of 85, 220, and 230 μm. After drying the electrode sheets for 2 h at 70 °C (non‐aqueous) or 60 °C (aqueous), they were calendered to estimated porosities of around 35 %. Circular electrodes were punched (Ø=14 mm) and the non‐aqueously processed electrodes were dried in a Büchi B‐585 glass drying oven under reduced pressure (<5×10^−2^ bar) at 120 °C for 16 h, the aqueous ones at 80 °C for 60 h. The average CAM mass loadings were (i) around 5.00±0.22 mg cm^−2^ (1.00±0.04 mAh cm^−2^) for investigations in NCM||Li metal cells and (ii) 11.9±0.8 mg cm^−2^ (2.38±0.16 mAh cm^−2^) for NCM||graphite full‐cell investigations. The areal capacities for the full‐cell investigations are based on the 2nd cycle discharge capacity from NCM||Li metal cells (at 20 mA g^−1^, 2.9–4.3 V).

The negative electrodes used for NCM||graphite full‐cell investigations consisted of 95 wt% commercial synthetic graphite (SMG−A5, Hitachi) as the active material, 1.5 wt% styrene‐butadiene‐rubber (SBR, SB5521, LIPATON; Polymer Latex GmbH) and 3.0 wt% sodium‐carboxymethyl cellulose (Na‐CMC, Walocel CRT 2000 PPA12, Dow Wolff Cellulosics) as binders, and 0.5 wt% carbon black as CA (Super C65, Imerys Graphite & Carbon). De‐ionized water was used as solvent for paste preparation. The paste viscosity was optimized to reach a solid content of around 40 wt% and homogenized as described above. The negative electrode paste was cast onto copper foil (10 μm, Nippon foil). After drying and calendering the graphite sheets to achieve 30 % porosity, Ø=15 mm circular electrodes were punched out, and the electrodes were dried in a Büchi B‐585 glass drying oven under reduced pressure (<5×10^−2^ bar) at 120 °C for 12 h. The average active mass loading of the negative electrodes was around 7±1 mg cm^−2^, resulting in an areal capacity of around 2.5±0.3 mAh cm^−2^ based on the practical capacity of graphite (≈350 mAh g^−1^) obtained from the 2^nd^ cycle discharge capacity from graphite||Li metal cells.

### Cell assembly and electrochemical characterization

All cells were assembled in dry room atmosphere with a dew point below −50 °C (relative humidity of 0.16 %). In order to validate reproducibility, three cells per sample were assembled. The standard deviation is represented by error bars in each figure. The specific current for a rate of 1 C was defined as 200 mA g^−1^ as obtained as practical specific discharge capacity (200 mAh g^−1^) in NCM||Li metal cells at 2.8–4.3 V.

The oxidative stability of the cathodes was evaluated using LSV. The investigations were performed in Swagelok cells with a three‐electrode setup.^[47]^ NCM electrodes with a smaller diameter (12 mm) were used as working electrode (WE) together with Li metal [lithium metal foil, 500 μm; battery grade: purity ≥99.9 %, China Energy Lithium (CEL Co.)] as reference (RE) and graphite (SMG−A5, Hitachi) as counter electrode (CE). Three layers of a polypropylene separator (FS 2190, Freudenberg Vliesstoffe SE & Co. KG) were used as separator and 1 m LiPF_6_ in 3 : 7 vol % ethylene carbonate (EC)/ethyl methyl carbonate (EMC) 3 : 7 as electrolyte (180 μL; battery line HTS; battery grade). The cells were tested for three cycles on the Maccor Battery tester 4000 at a C‐rate of 0.1 C between 2.8 and 4.2 V vs. Li|Li^+^. Afterwards, LSV measurements were performed at the Bio‐Logic VMP/VSP (Bio‐Logic SAS) at room temperature. For this, the potential of the WE was increased with a rate of 0.05 mV s^−1^ starting at the open circuit voltage and ending at 7.5 V vs. Li|Li^+^.

All other electrochemical investigations were carried out in a two‐electrode configuration^[47]^ in coin cells (CR2032, Hohsen Corporation). The C‐rate capability and long‐term cycling stability of Ni‐rich cathode materials were investigated in NCM||Li metal cells and NCM||graphite full‐cells, respectively, with 1 layer of a polymer membrane separator (diameter 16 mm, Celgard 2500) soaked in 1 m LiPF_6_ in 3 : 7 vol % EC/EMC 3 : 7 (35 μL; battery line HTS; battery grade) as electrolyte. At least three cells per sample were assembled to ensure a high reproducibility of our results. The standard deviation between cells is represented as error bars in the corresponding figures.

For NCM||Li metal cell investigations, Ni‐rich layered oxides as positive electrode (Ø14 mm; 1.00±0.04 mAh cm^−2^) and a Li metal negative electrode [Ø15 mm, lithium metal foil, 500 μm; battery grade: purity ≥99.9 %, China Energy Lithium (CEL Co.)] were used. For NCM||graphite full‐cell investigations, Ni‐rich layered oxides as positive electrode (Ø14 mm; 2.4±0.2 mAh cm^−2^) and graphite as negative electrode (Ø15 mm, 2.75±0.23 mAh cm^−2^) were considered. The negative/positive (N/P) capacity balancing ratio was set to 1.15 : 1.00 based on the 2^nd^ cycle discharge capacity from NCM||Li metal and graphite||Li metal cells investigations.

Electrochemical properties were investigated via constant‐current (CC) charge–discharge cycling on a Maccor Series 4000 battery tester (Maccor, Inc.) at 20 °C. The specific current for a rate of 1 C was defined as 200 mA g^−1^. The rate capability of cathode materials was investigated in NCM||Li metal cells according to the following procedure: 6 h at open‐circuit voltage (OCV) followed by two formation cycles at 0.1 C, three cycles at 0.2 C, and five cycles at 0.33, 0.5, 1, and 3 C each. For discharges rate above 0.2 C, asymmetric tests were performed, and the charge rate was kept to 0.2 C. The cell voltage window up to this point was 2.9 to 4.3 V. After the C‐rate investigations, cells were cycled at 0.1 C for two cycles, followed by 15 cycles at 0.33 C with an upper cut‐off cell voltage of 4.3 V.

The long‐term stability of cathode materials was evaluated in NCM||graphite full‐cells within the cell voltage range of 2.8–4.2 V. For that, these cells were cycled for four cycles at 0.1 C for effective interphase formation, followed by cycling at 0.33 C until dropping to 80 % SOH. Each 100^th^ cycle, cells were cycled at 0.1 C again for two cycles to evaluate the capacity retention. After each charge step, a constant‐voltage (CV) step was performed until the specific current reaches values ≤0.05 C.

## Conflict of interest

The authors declare no conflict of interest.

1

## Supporting information

As a service to our authors and readers, this journal provides supporting information supplied by the authors. Such materials are peer reviewed and may be re‐organized for online delivery, but are not copy‐edited or typeset. Technical support issues arising from supporting information (other than missing files) should be addressed to the authors.

Supporting Information

## Data Availability

The data that support the findings of this study are available from the corresponding author upon reasonable request.
