# Supplementary material for: Investigation of Lithium Polyacrylate Binders for Aqueous Processing of Ni‐Rich Lithium Layered Oxide Cathodes for Lithium‐Ion Batteries
Source: ChemSusChem. 2022 May 3;15(11):e202200401. doi: 10.1002/cssc.202200401 (PMC9321708; doi:10.1002/cssc.202200401)
Supplement: Supplementary file 1 — Supporting Information [file CSSC-15-0-s001.pdf]

ChemSusChem

Supporting Information

Investigation of Lithium Polyacrylate Binders for Aqueous Processing of Ni-Rich Lithium Layered Oxide Cathodes for Lithium-Ion Batteries

Friederike Reissig[+], Sebastian Puls[+], Tobias Placke, Martin Winter, Richard Schmuch,* and Aurora Gomez-Martin*© 2022 The Authors. ChemSusChem published by Wiley-VCH GmbH. This is an open access article under the terms of the Creative Commons Attribution License, which permits use, distribution and reproduction in any medium, provided the original work is properly cited.

Rheology studies of electrode pastes

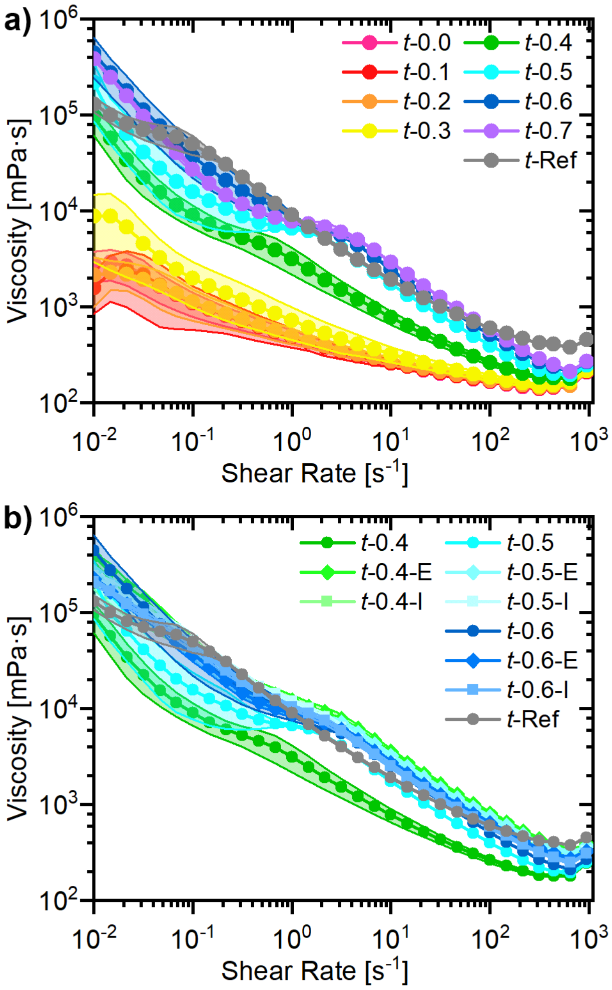

Figure S1: Rheological properties of selected positive electrode pastes. **a)** Impact of electrode pastes with various LiOH/PAA ratios prepared *via* the *Thinky Mixer* in comparison to PVdF/NMP pastes (*t*-Ref). **b)** Comparison of the impact of ethanol (*t*-__-E) and isopropanol (*t*-__-I) as co-solvents in comparison to the PVdF/NMP pastes (-Ref).

DSC analysis of LiPAA solutions after different processing methods

WILEY-VCH

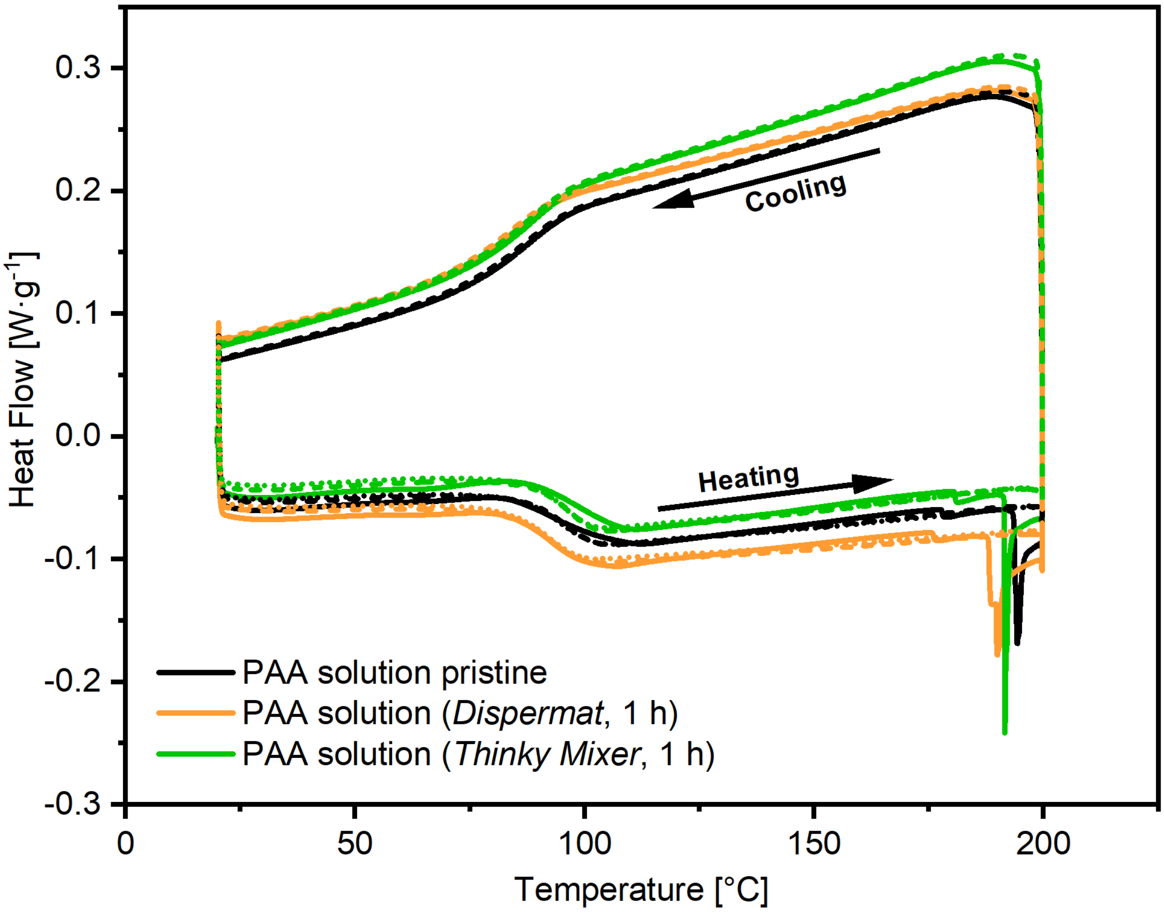

Figure S2: DSC curves of dried and differently treated PAA solutions. The samples were heated and cooled for 2.5 cycles with rates of 5 K min^{-1} to observe changes after the first cycle. The first cycle is marked by the solid, the second by the dashed and the third by the dotted line.

Table S1: Glass transition temperatures T_g for dried differently treated PAA solutions obtained from the DSC curves.

Sample	T_g (heating) [°C]			T_g (cooling) [°C]	
	1st cycle	2nd cycle	3rd cycle	1st cycle	2nd cycle
**PAA (pristine)**	93.6	95.5	94.6	88.4	87.9
**PAA (*Thinky Mixer*, 1 h)**	99.5	96.6	96.1	88.8	88.4
**PAA (*Dispermat*, 1 h)**	92.7	92.5	92.2	85.5	84.1

WILEY

SEM analysis of electrode coatings

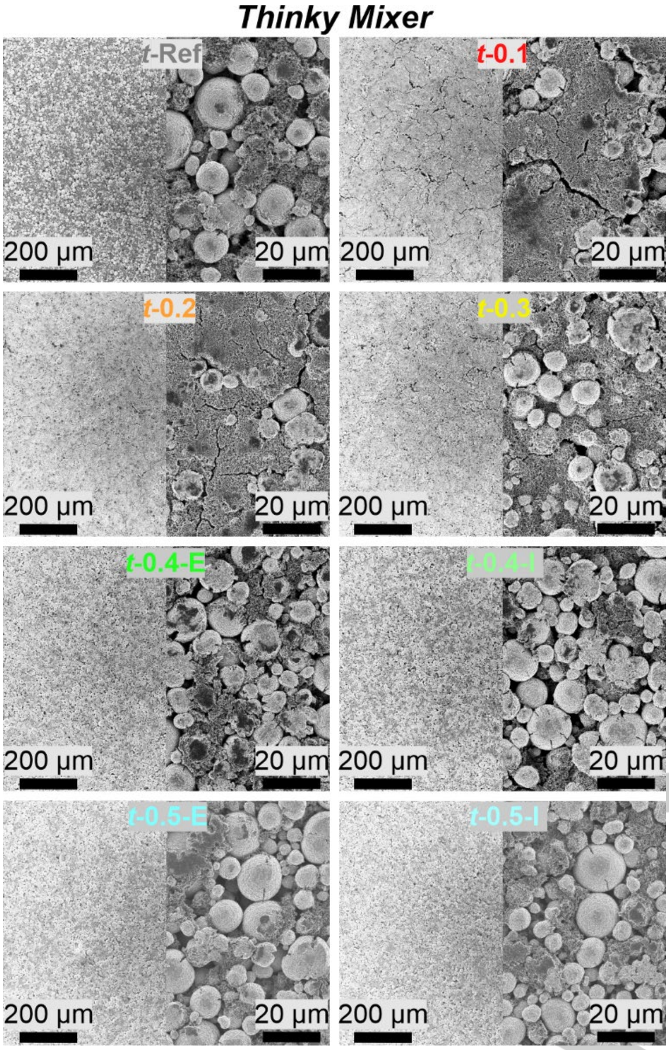

Figure S3: SEM images of composite cathodes prepared by different processing methods *(Thinky Mixer* (*t*-) and *Dispermat* (*d*-)) at two different magnifications. The samples are labeled with a small letter corresponding to the dispersing device ("*t*-" for *Thinky Mixer* and "*d*-" for *Dispermat*), followed by the molar ratio LiOH/PAA and optionally a capital letter for the co-solvent used. Two different magnifications are shown for each sample.

WILEY-VCH

Electrochemical Data

Table S2: Potential during LSV measurements at which a specific current of 0.02 mA g^{-1} is exceeded in a three-electrode setup with NCM electrodes as working electrode (WE), Li metal (lithium metal foil, 500 µm; battery grade: purity ≥99.9%, China Energy Lithium (CEL Co.)) as reference (RE) and graphite (SMG-A5, Hitachi) as counter electrode (CE). Given errors are the standard deviation of three cells for each sample. The samples are labeled with a small letter corresponding to the dispersing device ("*t*-" for *Thinky Mixer* and "*d*-" for *Dispermat*), followed by the molar ratio LiOH/PAA and optionally a capital letter for the co-solvent used.

Molar ratio LiOH/PAA	Potential *E* at which a specific current of 0.02 mA g^{-1} is exceeded *E* vs. Li|Li$^+$ /V			
	Thinky Mixer			*Dispermat*
	H_2O (*t*-__)	H_2O + EtOH (*t*-__-E)	H_2O + IPA (*t*-__-I)	H_2O (*d*-__)
**0.0**	5.18 ± 0.03	-	-	5.19 ± 0.02
**0.5**	5.19 ± 0.01	-	-	-
**0.6**	5.19 ± 0.03	5.216 ± 0.004	5.174 ± 0.002	5.19 ± 0.02
**0.7**	5.26 ± 0.06	-	-	5.33 ± 0.09
**NMP/PVdF-reference**	5.14 ± 0.01	-	-	5.14 ± 0.01

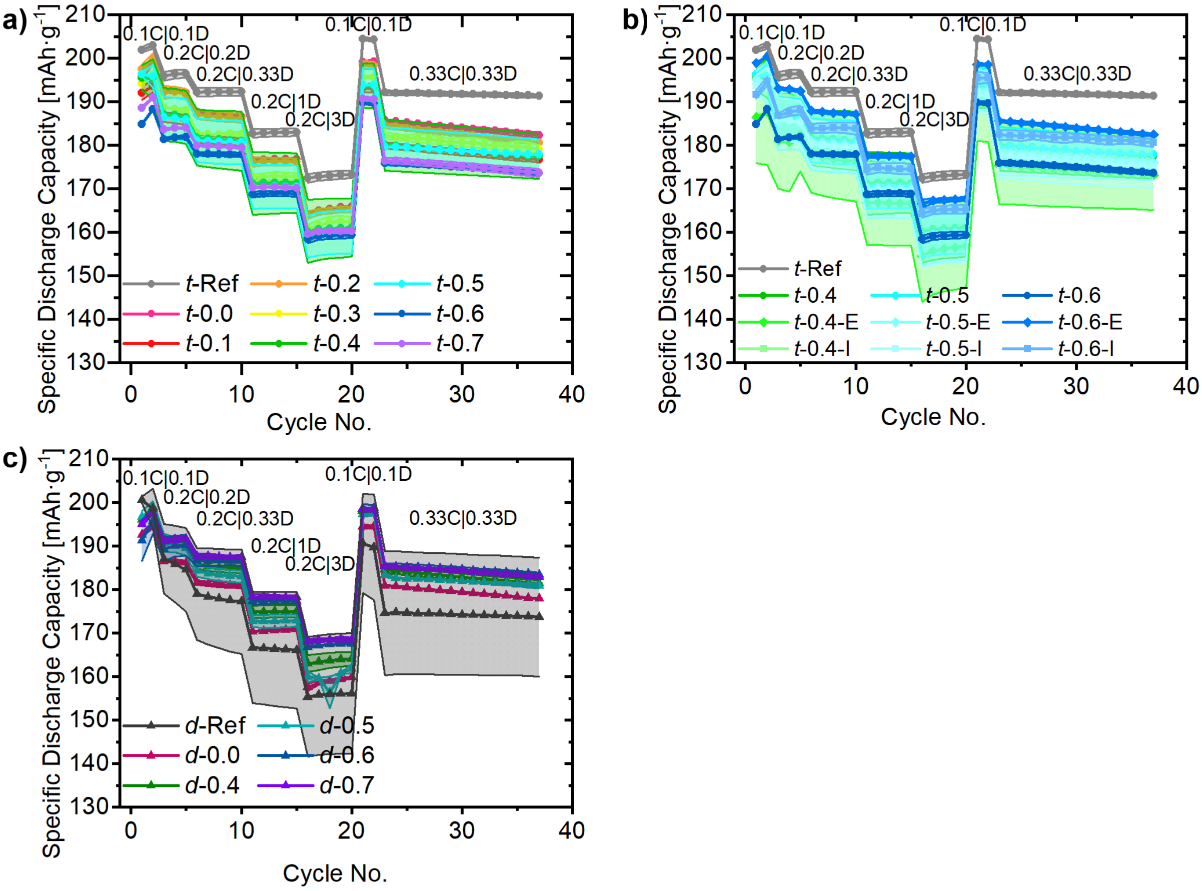

Figure S4: Rate capability investigations in NCM || Li metal cells (two-electrode configuration). a) specific discharge capacity vs. cycle number. Cell voltage range: 2.9 - 4.3V; 1C = 200 mA g^{-1}. Error bars: standard deviation of three cells for each sample. The samples are labeled with a small letter corresponding to the dispersing device ("*t*-" for *Thinky Mixer* and "*d*-" for *Dispermat*), followed by the molar ratio LiOH/PAA and optionally a capital letter for the co-solvent used.

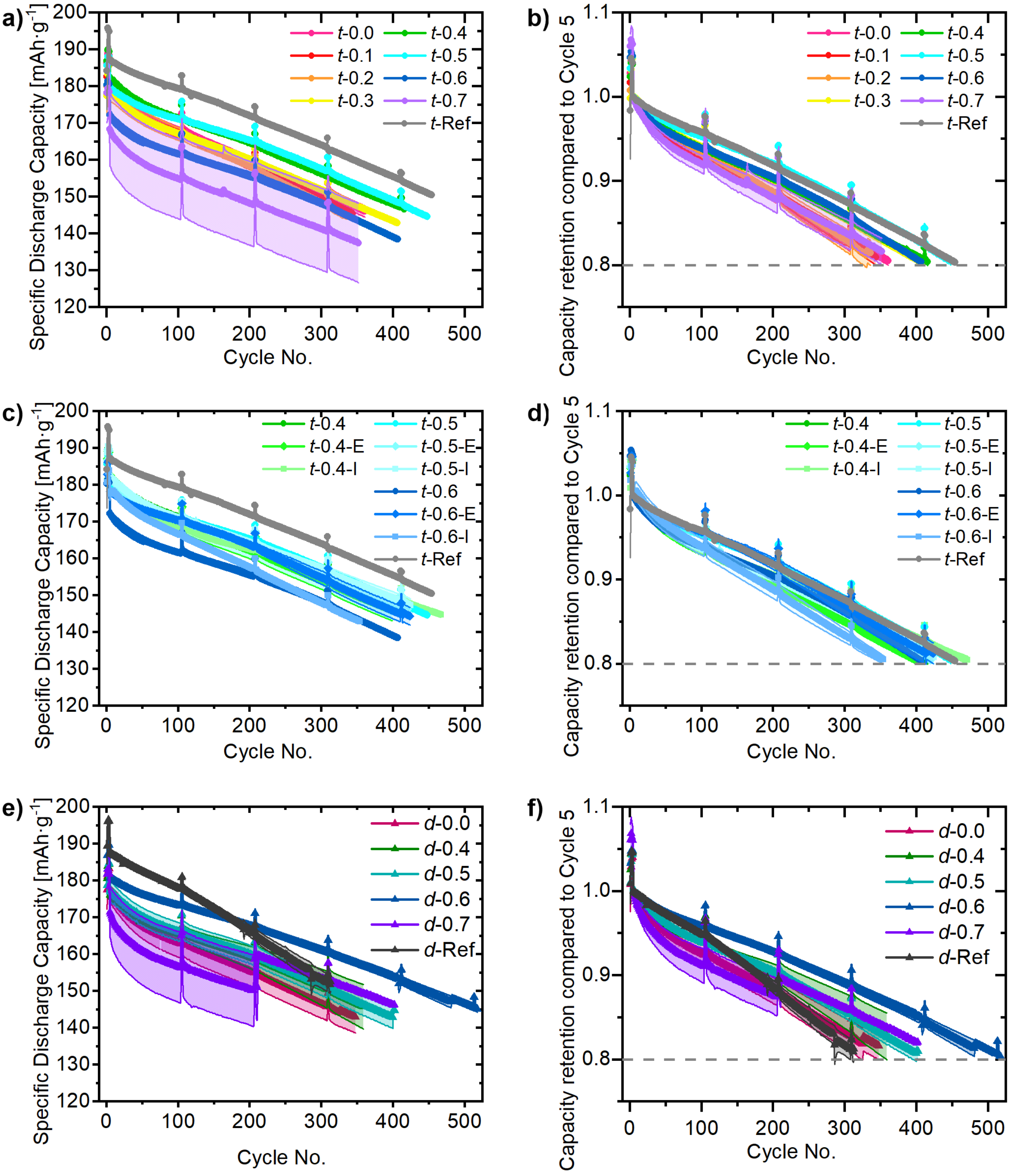

Figure S5: Results of electrochemical characterization in NCM || graphite full-cells (two-electrode configuration) with 4.2 V upper cut-off voltage. The first four cycles were conducted at 0.1C (1C=200 mA g^{-1}), while the following long-term cycling took place at 0.33C with two cycles at 0.1C each 100th cycle. **a), c), e)** Specific discharge capacities of all samples. **b), d), f)** Capacity retention to cycle 5 as SOH determination (80%) for all samples. The samples are labeled with a small letter corresponding to the dispersing device ("*t*-" for *Thinky Mixer* and "*d*-" for *Dispermat*), followed by the molar ratio LiOH/PAA and optionally a capital letter for the co-solvent used.

Table S3: Results of long-term cycling stability investigation in NCM || graphite full-cells. The given errors are the standard deviation of three cells per sample. The samples are labeled with a small letter corresponding to the dispersing device ("*t*-" for *Thinky Mixer* and "*d*-" for *Dispermat*), followed by the molar ratio LiOH/PAA and optionally a capital letter for the co-solvent used.

**Sample**	**Initial Coulombic efficiency [%]**	**Initial discharge capacity at 0.1 C after formation [mAh g^{-1}]**	**Initial discharge capacity at 0.33 C [mAh g^{-1}]**	**End of life (<80% SOH) reached in cycle:**
***t*-0.0**	84.7 ± 0.4	188.4 ± 0.7	181.3 ± 0.7	361
***t*-0.1**	84.28 ± 0.06	186.6 ± 0.2	179.6 ± 0.5	344
***t*-0.2**	84.6 ± 0.3	186 ± 2	179 ± 3	336
***t*-0.3**	81.9 ± 0.5	185.8 ± 0.1	177.70 ± 0.02	406
***t*-0.4**	84.4 ± 0.3	189.4 ± 0.4	182.4 ± 0.6	416
***t*-0.5**	84.1 ± 0.1	188.0 ± 0.1	179.6 ± 0.2	448
***t*-0.6**	85.0 ± 0.1	181 ± 1	172 ± 1	407
***t*-0.7**	85 ± 1	178 ± 8	170 ± 10	352
***t*-Ref**	85.9 ± 0.6	194.7 ± 0.5	187.3 ± 0.3	454
***t*-0.4-E**	84 ± 1	189 ± 2	181 ± 2	399
***t*-0.4-I**	82.8 ± 0.4	187.1 ± 0.3	179.62 ± 0.07	471
***t*-0.5-E**	84.6 ± 0.1	189.25 ± 0.09	181.7 ± 0.2	420
***t*-0.5-I**	84.78 ± 0.08	190.0 ± 0.5	182.3 ± 0.5	425
***t*-0.6-E**	84.1 ± 0.1	186.2 ± 0.2	178.1 ± 0.6	424
***t*-0.6-I**	83.8 ± 0.4	183.9 ± 0.7	177 ± 2	354
***d*-0.0**	83 ± 1	183 ± 2	176 ± 2	348
***d*-0.4**	82.4 ± 0.5	184 ± 1	176 ± 1	359
***d*-0.5**	83 ± 1	185 ± 2	177 ± 3	403
***d*-0.6**	84.7 ± 0.2	189.5 ± 0.5	180.8 ± 0.5	518
***d*-0.7**	84.5 ± 0.6	182 ± 4	171 ± 7	402
***d*-Ref**	86.8 ± 0.3	196.1 ± 0.3	187.6 ± 0.2	313

WILEY-VCH

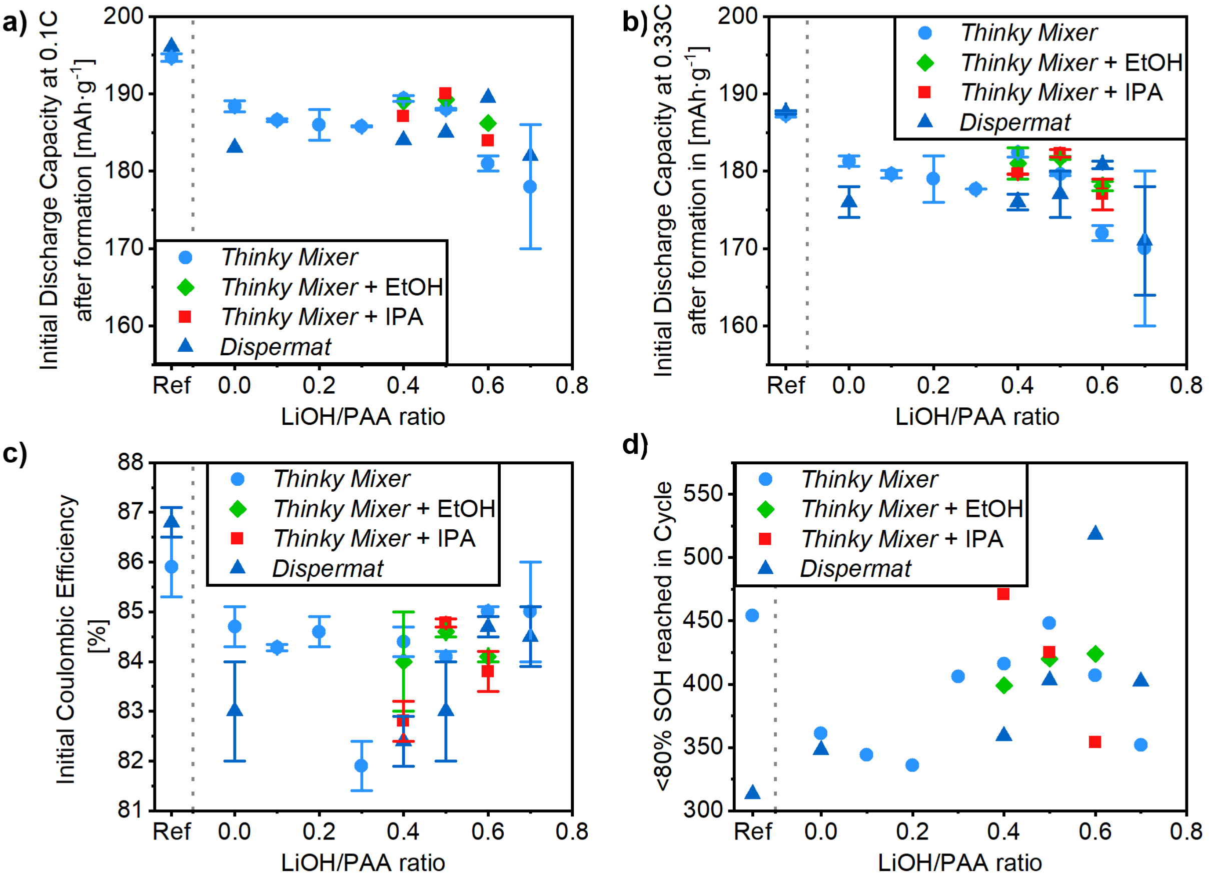

Figure S6: Results of electrochemical characterization in NCM || graphite full-cells with 4.2 V upper cut-off voltage. The first four cycles were conducted at 0.1C (1C=200 mA g^{-1}), while the following long-term cycling took place at 0.33C with two cycles at 0.1C each 100th cycle. **a)** Initial discharge capacities at 0.1C after formation (cycle 4) *versus* the LiOH/PAA ratio with the PVdF/NMP references (Ref) in comparison. **b)** Initial discharge capacities at 0.33C after formation (in cycle 5) *versus* the LiOH/PAA ratio with the PVdF/NMP references (Ref) in comparison. **c)** Initial Coulombic efficiency in the first cycle *versus* the LiOH/PAA ratio with the PVdF/NMP references (Ref) in comparison. **d)** Cycle in which the end-of-life criterion of 80 % state-of-health (SOH) is reached *versus* the LiOH/PAA ratio with the PVdF/NMP references (Ref) in comparison. The samples are labeled with a small letter corresponding to the dispersing device ("*t*-" for *Thinky Mixer* and "*d*-" for *Dispermat*), followed by the molar ratio LiOH/PAA and optionally a capital letter for the co-solvent used.

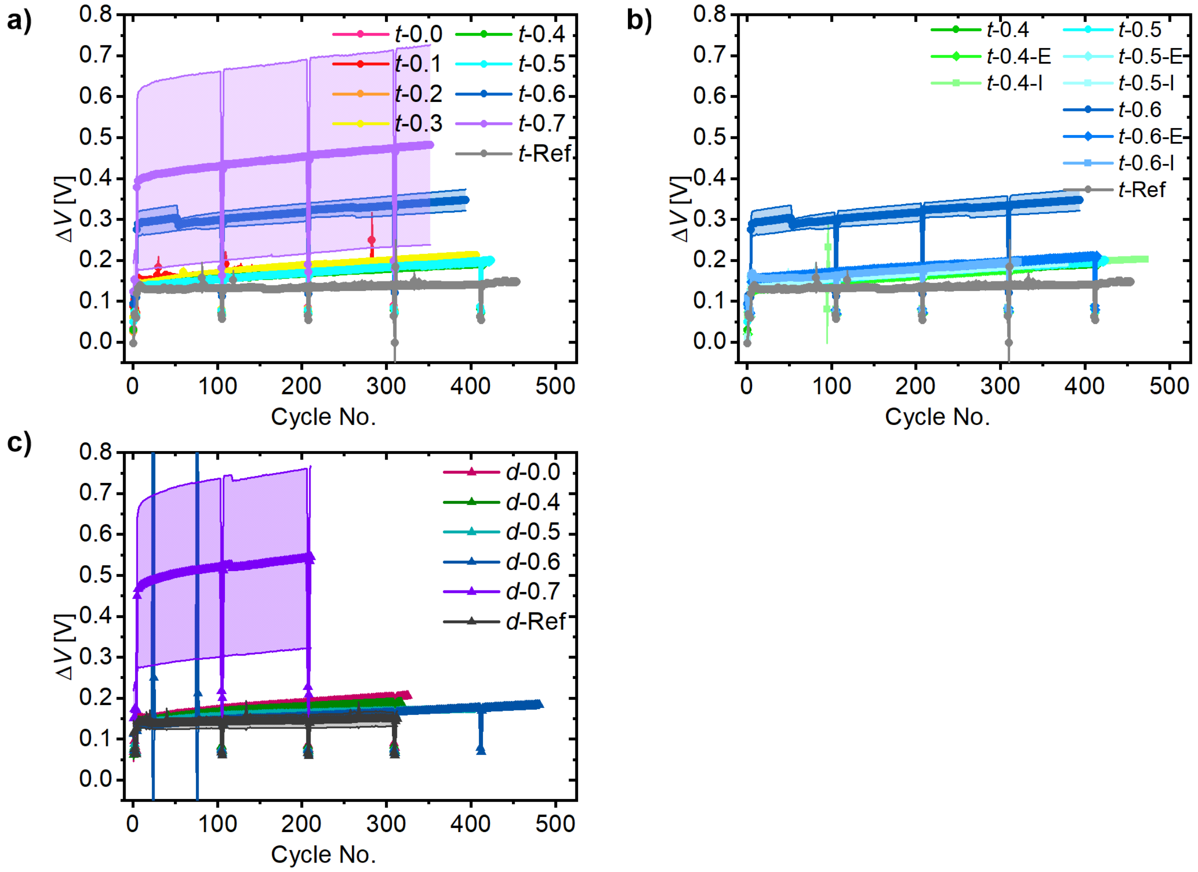

Figure S7: Polarization growth (ΔV= $V_{average,charge}$ – $V_{average,discharg}$e) during electrochemical cycling characterization in NCM || graphite full-cells with 4.2 V as upper cut-off voltage. The first four cycles were conducted at 0.1C (1C=200 mA g^{-1}), while the following long-term cycling took place at 0.33C with two cycles at 0.1C each 100th cycle. The samples are labeled with a small letter corresponding to the dispersing device ("*t*-" for *Thinky Mixer* and "*d*-" for *Dispermat*), followed by the molar ratio LiOH/PAA and optionally a capital letter for the co-solvent used.

WILEY
